# Integrating phylogenetic and functional data in microbiome studies

**DOI:** 10.1093/bioinformatics/btac655

**Published:** 2022-09-30

**Authors:** Gavin M Douglas, Molly G Hayes, Morgan G I Langille, Elhanan Borenstein

**Affiliations:** Department of Microbiology and Immunology, McGill University, Montréal, QC H3A 2B4, Canada; Department of Mathematics and Statistics, Dalhousie University, Halifax, NS B3H 4R2, Canada; Department of Microbiology and Immunology, Dalhousie University, Halifax, NS B3H 4R2, Canada; Blavatnik School of Computer Science, Tel Aviv University, Tel Aviv 6997801, Israel; Department of Clinical Microbiology and Immunology, Sackler Faculty of Medicine, Tel Aviv University, Tel Aviv 6997801, Israel; Santa Fe Institute, Santa Fe, NM 87501, USA

## Abstract

**Motivation:**

Microbiome functional data are frequently analyzed to identify associations between microbial functions (e.g. genes) and sample groups of interest. However, it is challenging to distinguish between different possible explanations for variation in community-wide functional profiles by considering functions alone. To help address this problem, we have developed *POMS*, a package that implements multiple phylogeny-aware frameworks to more robustly identify enriched functions.

**Results:**

The key contribution is an extended balance-tree workflow that incorporates functional and taxonomic information to identify functions that are consistently enriched in sample groups across independent taxonomic lineages. Our package also includes a workflow for running phylogenetic regression. Based on simulated data we demonstrate that these approaches more accurately identify gene families that confer a selective advantage compared with commonly used tools. We also show that POMS in particular can identify enriched functions in real-world metagenomics datasets that are potential targets of strong selection on multiple members of the microbiome.

**Availability and implementation:**

These workflows are freely available in the *POMS* R package at https://github.com/gavinmdouglas/POMS.

**Supplementary information:**

Supplementary data are available at *Bioinformatics* online.

## 1 Introduction

Microbiome sequencing has been applied to characterize myriad environments and is typically analyzed based on the relative abundance of microbial features. These features may include both taxa (the microbes present) and functions (the genes and pathways they encode). While both data types have been leveraged to make valuable observations, they are typically analyzed independently. Yet, linking these data types is required to make coherent interpretations of observed shifts in microbiome data (Douglas and Langille, 2021; Manor and Borenstein, 2017). For example, an enrichment of a particular microbial pathway in a group of samples (e.g. a disease or unique environment) could represent selection on multiple independent taxa which possess that pathway, which would be of significant biological interest. In contrast, such an enrichment could have also arisen due to the presence of that pathway in a specific microbial taxon that is more abundant for various other reasons in the group of interest, making this enrichment less biologically interesting.

Two approaches have been developed that partially address the challenge of how to link taxonomic and functional data types. *FishTaco* (Manor and Borenstein, 2017) is a tool that pinpoints the taxonomic contributors to functional shifts identified by differential abundance tests. *FishTaco*, however, is a post-hoc tool that is applied after significant microbial functions have been identified, whereas ideally, functional and taxonomic data would be integrated while testing for differential functions to better identify strong enrichment candidates.

*Phylogenize* is an approach that explicitly integrates functional and taxonomic data during statistical testing (Bradley *et al.*, 2018; Bradley and Pollard, 2020). It identifies functional associations based on the prevalence or specificity of taxa that encode each gene family. This is performed using phylogenetic linear models, which account for the genetic similarity of co-occurring taxa that arises due to their shared evolutionary history. Using this framework, significant gene families and pathways that are contributed by a diverse set of taxa from within a given phylum are identified. Although this approach forms a key improvement over past analytical approaches, it has not been widely adopted. This is at least partially because *phylogenize* requires that users limit their analyses to genomes in the MIDAS database (Nayfach *et al.*, 2016).

Some of the complexity of integrating taxonomic and functional analyses, as well as other challenges in microbiome data analysis, stem from the difficulties of applying standard statistical approaches to raw microbiome data due to their compositional nature. Fortunately, there is growing interest in improved compositional approaches for analyzing microbiome data. Specifically, analyzing ratios of microbiome feature relative abundances (rather than the abundances of features themselves) has recently been proposed as a solution to the compositionality problem (Gloor *et al.*, 2016; Morton *et al.*, 2019). However, it is unclear which features should be used for stably computing these ratios. One proposed solution is to compare ratios of taxa (based on the isometric log-ratio transformation) on each side of every node in a phylogenetic tree that links the various taxa (Silverman *et al.*, 2017). This general approach is now commonly referred to as analyzing balance trees (Morton *et al.*, 2017). While this is a statistically valid framework for analyzing microbiome data, it is often unclear how to interpret differences in taxonomic ratios.

Herein, we aim to address both the challenges involved in functional differential abundance analysis and the limited interpretability of balance tree-based approaches by testing for functional enrichment in a balance tree framework. Specifically, our approach, termed Phylogenetic Organization of Metagenomic Signals (POMS), focuses on identifying cases where multiple taxa that encode a given function are consistently associated with a sample group. Such cases provide more support to the hypothesis that the function itself confers a selective advantage to microbes in the relevant sample group, rather than that the function happens to be present in just a few taxa that are more abundant in that group. In addition to the POMS workflow, we have also re-implemented several functions present in *phylogenize*, which allows this phylogenetic regression framework to be applied to novel genomes. We find that each workflow pinpoints interesting functional associations in both simulated and real metagenomics data, and that they have complementary strengths and weaknesses. This is a valuable proof-of-concept that integrating functional enrichments into balance tree analyses improves their interpretability and provides novel insights. Importantly, however, since POMS cannot identify functions with limited taxonomic breadth as highly enriched, we see it primarily as a complementary tool to phylogenetic regression, and other more sensitive approaches.

## 2 Materials and methods

### 2.1 Isometric log-ratio

Phylogenetic balances in POMS are calculated based on the isometric log-ratio of taxa in the two node subtrees (i.e. on one side of the node compared to the other; Morton *et al.*, 2017; Silverman *et al.*, 2017). This approach converts microbiome relative abundance data into ratios of geometric means. Specifically, the balance for a sample at node *i* is calculated based on the equation

$$b_{i}=\sqrt{\frac{n_{Li}n_{Ri}}{n_{Li}+n_{Ri}}}\;\text{log}\;\frac{g(y_{Li})}{g(y_{Ri})},$$

where $n_{Li}$ and $n_{Ri}$ correspond to the numbers of taxa on the left- and right-hand sides of the node. Similarly, $g(y_{Li})$ and $g\left(y_{Ri}\right)$ correspond to the geometric means of the relative abundances of taxa on the left- and right-hand sides of the node. Note that the choice of which lineage is considered the left- versus right-hand side of a given node is arbitrary. The numbers of taxa on each side of a node are included in this calculation to scale the balance to give it unit length (i.e. to make the balances comparable despite varying numbers of taxa at each node).

The geometric mean of the relative abundance of a set of taxa on the left-hand side is calculated based on the equation

$$g\left(y_{Li}\right)={\left(\prod_{j=1}^{n_{Li}}y_{j}\right)}^{\frac{1}{n_{Li}}}=\sqrt[{n_{Li}}]{y_{1}y_{2}\ldots y_{n_{Li}}},$$

and analogously for the right-hand side. If any taxa have a relative abundance of 0 then the geometric mean will also be 0. To avoid this issue, a pseudocount can be added prior to computing these values. For all POMS analyses in this manuscript, we added a pseudocount of 1 to taxonomic abundances prior to computing the isometric log-ratios.

### 2.2 Multinomial test

A multinomial exact test is used in POMS to identify consistently enriched functions (CEFs), which is described in Section 3. This test considers the counts of three classes of function-significant nodes (FSNs) per function: (i) FSNs that do not intersect with balance-significant nodes (BSNs), (ii) FSNs that intersect with BSNs where the functional enrichment is in taxa that are relatively more abundant in sample group one and (iii) FSNs that intersect with BSNs where the functional enrichment is in taxa that are relatively more abundant in sample group two. The null expectation for the proportion of FSNs in each category is based on a mass-action interaction between FSNs and BSNs. In other words, the null expectation corresponds to the case where FSNs and BSNs are assigned randomly. The expectation also assumes that FSNs that intersect with BSNs are split equally between BSNs that are higher in sample groups one and two, respectively. This test is implemented with the xmulti function of the *XNomial* R package (tested with version 1.0.4), using the default log-likelihood ratio statistic to compute the *P*-value

### 2.3 Phylogenetic regression

We performed phylogenetic regression using the phylolm function from the *phylolm* (Ho and Ané, 2014) R package, which regresses a vector against the copy number of each tested function, while taking the phylogenetic similarity of tips into account. The default Brownian motion model was used for all analyses. We implemented functions in the *POMS* package for running this process, as well as for computing the prevalence and specificity scores described as part of *phylogenize*. These latter functions were adapted from the *phylogenize* v0.94 codebase (https://bitbucket.org/pbradz/phylogenize), which is distributed under an MIT license.

### 2.4 Pre-processing function copy-number tables

The same filtering cutoffs were used to filter the function copy number tables ([which link metagenome-assembled genomes (MAGs) and other taxa to genome annotations]) prior to running all analyses. First, the tables were restricted to include only taxa present in at least one sample used for the relevant analysis. Then, any function found in fewer than five taxa, or <0.1% of taxa, was removed.

### 2.5 MAG-based simulations

The MAG-based simulations were based on 704 control samples from a large human meta-analysis dataset (Almeida *et al.*, 2019). These samples all met the metadata criteria to be labeled as derived from healthy adults not on antibiotics. These samples were also from studies that included at least 40 samples in total. One sample was randomly dropped to create balanced groups. MAG abundance was taken as the previously computed mean read depth (i.e. mean number of mapped reads per site). MAGs with a breadth of coverage less than 25% in a sample were given a mean depth of 0 in that sample. We then excluded all MAGs that were not called as present in any sample, leaving 1595 MAGs remaining for the simulations.

These simulations proceeded as described in Section 3. First, the samples were randomly split into two groups for each of the 1000 replicate datasets. Then, for each profile a focal gene family was randomly chosen, and within one group the abundance of all MAGs encoding this gene family was incremented by a pseudocount of 1 and these abundances were multiplied by a factor of 1.5. These simulated datasets are referred to as the ‘focal gene’ profiles. We also performed parallel simulations where the relative abundance of random taxa was randomly inflated by identical amounts in one group only. Importantly, the same number of taxa were perturbed as were affected in each matching focal gene simulation profile. The resulting simulated profiles are referred to as the ‘random taxa’ profiles. The ‘clade-based’ profiles represent an additional set of simulations that use the same selective pressures but applied only to taxa descendent from a given node in the phylogenetic tree. There is one clade-based dataset for each of the 693 nodes in the test tree with at least five underlying taxa (excluding the root node).

We performed two types of phylogenetic regression on these simulated datasets. In the first, the model response was the specificity of taxa to the first sample group. In the second, the model response was a binary indicator of whether taxa significantly differed in relative abundance between the sample groups, based on Wilcoxon tests (uncorrected *P* < 0.05). The alternative differential abundance approaches compared in this study were Wilcoxon tests following normalization by the median universal single-copy gene (USCG) abundance (using the same approach used by the tool MUSiCC; Manor and Borenstein, 2015), Wilcoxon tests based on relative abundances, ALDEx2 v1.16.0 (Fernandes *et al.*, 2014), DESeq2 v1.24.0 (Love *et al.*, 2014) and limma-voom v3.40.6 (Law *et al.*, 2014; Ritchie *et al.*, 2015). Significant gene families were identified based on a Benjamini–Hochberg cutoff of <0.05 for all tested approaches, including POMS.

### 2.6 Tara oceans dataset validation

The assembled Tara Oceans metagenomics dataset was taken from a pre-existing project (Delmont *et al.*, 2018). Environmental and chemical profiles of the ocean samples along with the relative abundance and annotations of MAGs were taken from the published Supplementary Tables for this project.

GToTree v1.4.16 (Lee and Ponty, 2019) was run to build a phylogenetic tree based on shared single-copy genes and to exclude MAGs with completeness below 60% and redundancy above 10%, which resulted in retaining 642 MAGs. MAGs were called as present within samples if the breadth of coverage was >1%. This is a lenient setting, which was chosen based on the empirical distributions of MAG breadth of coverage across the samples.

Higher-level functions (i.e. KEGG modules and pathways) were reconstructed based on KEGG mappings from KOs to these categories downloaded from the KEGG website (Kanehisa *et al.*, 2016) on April 12, 2021. Reconstruction was performed using the *PICRUSt2* script pathway_pipeline.py (Douglas *et al.* 2020), which leverages *MinPath* (Ye and Doak, 2009) and the algorithm implemented in *HUMAnN2* (Franzosa *et al.*, 2018) to reconstruct higher-level function abundances. These reconstructions were performed for each MAG independently.

Because the environmental data, such as the salinity and nutrient concentrations, were continuous, significant BSNs were identified based on Spearman correlations between sample balances and these environmental factors (uncorrected *P* < 0.05). These results were then discretized to indicate whether the factors were positively or negatively associated with the groups at each respective BSN. Phylogenetic regression was conducted based on a binary indicator of whether taxon abundances showed significant Spearman correlation (uncorrected *P* < 0.05) with each environmental factor. Spearman correlations were also computed based on the function relative abundances themselves against the environmental factors. The cross-product of the taxonomic abundances per sample and the functional abundances per taxa was used to produce the community-wide functional abundances.

Finally, Faith’s phylogenetic diversity was computed based on the subset of taxa encoding each significant hit output by POMS or phylogenetic regression. This was done using the *Picante* R package (v1.8.2; Kembel *et al.*, 2010).

### 2.7 Case–control shotgun metagenomics dataset validations

We focused certain validation analyses on three datasets that were part of a large meta-analysis of human shotgun metagenomics datasets (Almeida *et al.*, 2019). These datasets are defined based on dataset accession identifiers in the European Nucleotide Archive. We used the previously generated MAGs, sample MAG abundance profiles and MAG phylogenetic tree as inputs to POMS after performing the same pre-processing steps that were performed for the MAG-based simulations, but on a distinct subset of samples. Phylogenetic regression was conducted based on the specificity of each taxon for samples in the case group for each dataset. Faith’s phylogenetic diversity computation and reconstruction of KEGG pathways and modules was performed as for the Tara Oceans dataset.

### 2.8 POMS dependencies

*POMS* is written in R (R Core Team, 2019) and is dependent on the following R packages (versions used in this manuscript are indicated, but these exact versions are not required): *ape* v5.3 (Paradis *et al.*, 2004), *parallel* v3.6.0, *phangorn* v2.5.5 (Schliep, 2011), *phylolm* v2.6.4, *stringr* v1.4.0 and *XNomial* v1.0.4. *POMS* v0.3.1 was used for all analyses. Testing and development of this approach was carried out using R v3.6.0 and RStudio v1.2.5033 on a server running Ubuntu v16.04.5.

Several additional R packages are required to follow the current analysis workflow after running *POMS* (again the versions indicated were used for this article, but are not required versions): *ggtree* (Yu, 2020; Yu *et al.*, 2017) v1.16.1, *ggplot2* v3.3.0 (Wickham, 2016), *plyr* v1.8.4 (Wickham, 2011) and *reshape2* v1.4.3 (Wickham, 2007). All multi-panel plots displayed were created with the *cowplot* (v1.0.0) R package.

### 2.9 Code availability

The POMS source code is available at: https://github.com/gavinmdouglas/POMS. The code for all analyses presented in this manuscript is available at: https://github.com/gavinmdouglas/POMS_manuscript/.

## 3 Results

### 3.1 POMS overview

**Fig. 1.**
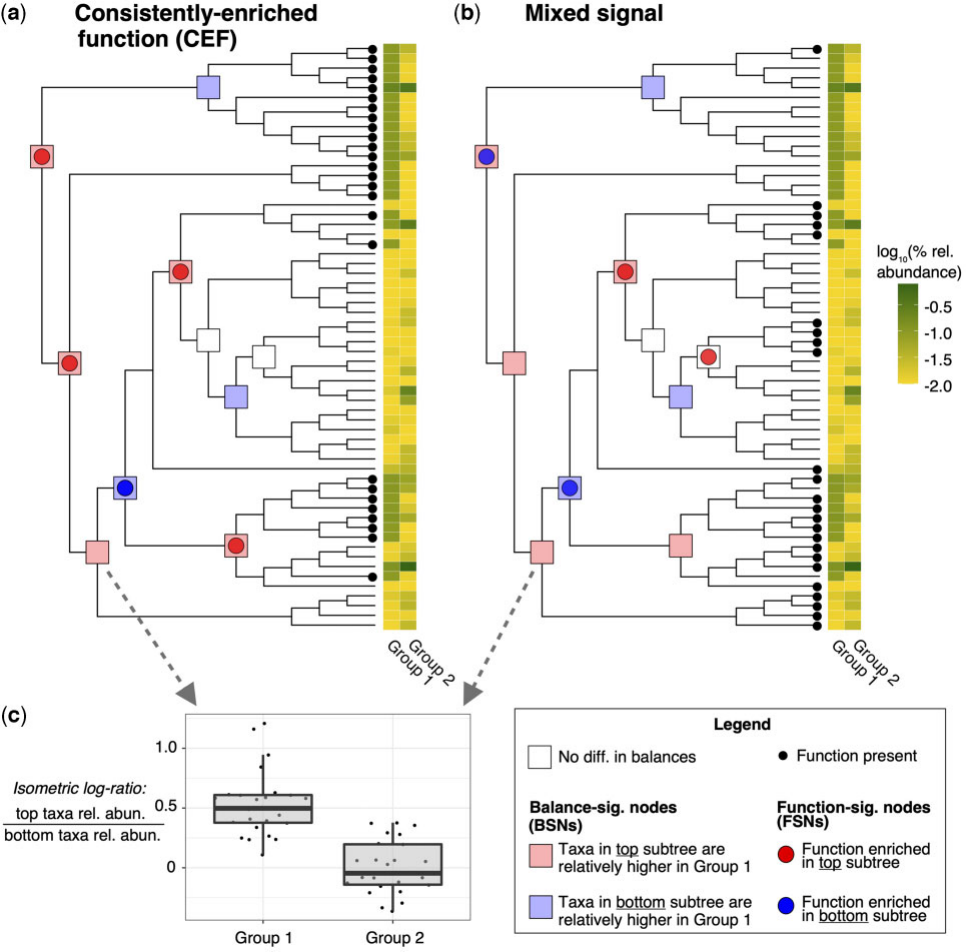
Contrasting examples that illustrate the POMS methodology. (**a** and **b**) The two phylogenetic trees correspond to 60 microbial genomes found across two sample groups (Groups 1 and 2). The mean log_10_ relative abundance of these genomes in each group is indicated by the heatmaps. The squares indicated on tested nodes indicate whether there is a difference in the isometric log-ratio of the subtree taxonomic relative abundances between the two sample groups. Red and blue squares are colored to indicate which subtree is at relatively higher levels in Group 1 samples (relative to the other subtree; see panel **c** for an example). Note that the taxonomy-based annotations are identical between the two panels. Panels a and b show the distribution of two different microbial functions across these genomes (small black dots at the tips of trees). The colored circles on the tested nodes indicate that the function of interest is enriched in one subtree compared with the other. POMS tests whether the intersection between colored squares and circles is higher than expected by chance. Functions that are consistently enriched in the sample group direction (i.e. where the circle and square colors match at nodes consistently, as in panel a, or alternatively where they consistently mismatch) are particularly of interest. Panel b is a contrasting example where the function is not consistently enriched in taxa relatively higher in the same sample group. Note that the minimum number of underlying tips on each side for a node to be retained for analysis was set to four for this example, but it would normally be higher (A color version of this figure appears in the online version of this article.)

POMS is a balance tree framework for analyzing microbial functions (Fig. 1), including both gene families and higher-level functions. The key input tables correspond to taxonomic abundances across samples and per-taxon functional abundances. A phylogenetic tree containing all taxa present in the samples must also be provided. The per-taxon functional abundance table corresponds to genome annotations for MAGs, or other known taxa present in an environment. This format contrasts with the functional abundance tables that are largely unlinked from specific taxa, such as those that are produced through read-mapping against a database of broadly distributed gene families. The key POMS output is a table summarizing, for each annotated function, the results of the test for consistent enrichment.

The workflow begins by first identifying all nodes in the tree with sufficient underlying tips (10 by default) on both the left- and right-hand sides. All nodes that do not fit these criteria are excluded from the analysis. The balances of taxa (per sample) at the remaining nodes, defined as the isometric log-ratios of the relative abundances of taxa on the left-hand side compared with those on the right-hand side, are then computed. Typically, a pseudocount is added to the taxonomic abundances to account for cases where taxa are absent. Standard statistical tests can then be used to determine whether these phylogenetic balances differ between sample groups. Put simply, a node with significantly different balances between sample groups indicates that the taxa on either the left- or right-hand side of that node are at significantly higher abundances relative to the taxa on the other side of the node in one sample group compared with the other. Significantly different balances between sample groups are identified based on Wilcoxon rank-sum tests (uncorrected *P* < 0.05 by default). Alternatively, the user can specify which nodes are significantly different based on an external, user-defined test, which enables more customized analyses.

We refer to each node with a significantly different balance as a BSN. After identifying each BSN, POMS further determines which side of the node is enriched within each sample group. In other words, POMS determines which taxa are at relatively higher levels (relative to taxa on the other side of the node) in each sample group. Notably, such taxa are not necessarily at higher relative abundances in one group or the other, but rather the relative ratios of the taxa on each side of the node are different between the sample groups.

Next, POMS tests for enrichment of each annotated function across the nodes. More specifically, a Fisher’s exact test is computed based on the counts of tips that either do or do not encode the function on either side of the node (uncorrected *P* < 0.05 by default). Importantly, these enrichment tests are computed at all nodes tested during the balance tree step: not just at BSNs. We refer to each significant node based on this approach as a FSN. Again, put simply, an FSN indicates that the tested function is enriched in taxa on either side of the node compared with the other.

Finally, a multinomial exact test is applied to test whether FSNs coincide with BSNs, and consistently in the direction of the same sample group, more often than expected by chance. Specifically, for each tested function, the set of identified FSNs for that function (i.e. nodes at which this function is enriched in one side of the node compared to the other) can be partitioned into three classes. The first class corresponds to FSNs that do not intersect with BSNs (i.e. there is a significant enrichment of a function on one side of the node, but there is no significant difference in sample balances). The other two classes correspond to FSNs that intersect with BSNs: one class where the function is enriched in taxa that are relatively more abundant in the first sample group, and the other class where the function is enriched in taxa that are relatively more abundant in the second sample group. In our example in Figure 1 these two classes are represented by intersecting circles and squares of the same and different colors, respectively. For each function, the multinomial exact test is performed based on the number of FSNs in each class compared with the expected proportions given random intersections between FSNs and BSNs. We refer to significant functions based on this test as CEFs.

Although this test is primarily intended to identify CEFs enriched in the direction of a single sample group, this framework can also identify CEFs that show mixed signal of enrichment toward both sample groups. In other words, a function could be significant because there is a depletion of FSNs that do not intersect with BSNs compared with the random expectation, and not that it is consistently enriched toward a particular sample group. Such cases could still be biologically interesting, but this highlights that post-hoc consideration of the number of FSNs of each class is needed to be able to interpret CEFs appropriately.

### 3.2 Simulation-based validations

To validate POMS, we first generated simulated datasets based on samples containing MAGs. The MAGs from shotgun metagenomics (MGS) control samples, with corresponding mean depths and phylogenetic tree, were obtained from a large human gut MGS meta-analysis. These MAGs had previously been annotated with KEGG orthologs (KOs). We subsampled 704 control samples into two equally sized groups 1000 times to create random test datasets.

We then conducted several sets of simulations to evaluate POMS's performance. For all these simulations we compared POMS’s performance with our implementation of phylogenetic regression. This regression was based on either significant taxa between the groups as determined by Wilcoxon tests, or on the specificity score developed as part of *phylogenize.* Although this comparison provides insight into how POMS compares to an existing methodology that similarly integrates taxonomic and functional information, the more practical question is how these methods perform compared to standard differential abundance tools, as the latter are more commonly applied. Accordingly, we also ran several differential abundance tools, including ALDEx2, DESeq2, limma-voom and Wilcoxon tests (based on raw relative abundances or corrected by the abundance of USCGs). We applied these standard differential abundance approaches to KO abundance tables that lacked taxonomic links. The per-sample abundance of each KO in these tables was computed by summing the product of the taxon abundance and KO copy number for each taxon that encoded the KO, i.e. we computed the cross-product of the taxa abundance and KO copy number tables.

To characterize the baseline behavior of each tool, we first investigated the proportion of significant KOs [based on Benjamini–Hochberg corrected *P*-values (BH) < 0.05] identified by each tool across the 1000 random datasets. Some tools identified significant KOs only in a small number of datasets; POMS identified significant KOs in 12 datasets, while ALDEx2 and the relative abundance and USCG-corrected Wilcoxon test approaches identified 7, 17 and 19 datasets with significant KOs, respectively. In contrast, DESeq2, limma-voom and the significance-based and specificity-based phylogenetic regression approaches identified significant KOs in 543, 1000, 1000 and 1000 datasets, respectively. Although three tools called KOs as significant in all datasets, the proportion of significant KOs per-dataset was low. The mean proportion of significant KOs was 0.163 [standard deviation (SD) = 0.038] for limma-voom, 0.060 (SD = 0.029) for the significance-based phylogenetic regression, and 0.056 (SD = 0.027) for the specificity-based phylogenetic regression.

We then introduced taxonomic variation between the two groups in each dataset using three different approaches. In the first approach, for each of the 1000 random datasets, we randomly selected a KO encoded by at least five MAGs. We then simulated selection acting upon the genomes encoding this gene, by adding a pseudocount of one to these genomes and then multiplying their abundance by 1.5 in one sample group only (Supplementary Fig. S1). Each randomly selected gene is referred to as the focal gene per dataset and represents a gene that confers a selective advantage in one of the two sample groups to taxa that encode it. This set of simulated datasets is referred to as the focal gene profiles. In the second approach we conducted analogous simulations, but where random genomes (rather than genomes that encode a specific focal gene) were selected to increase in abundance in one group. For consistency, the number of random genomes that were perturbed in this approach in each profile was the same as the number of genomes encoding the focal gene in the corresponding focal gene profile. This set of simulated datasets is referred to as the random taxa profiles. Finally, we also simulated cases where all taxa in the same clade, rather than randomly selected taxa, were perturbed. This involved perturbing the abundance of taxa underlying each node (for all nodes with at least underlying five tips and excluding the root node) in the tree. This resulted in 693 datasets, corresponding to each such node in the tree. These datasets are referred to as the clade-based profiles. We included these simulations to explore to what degree POMS is robust to the blooming of individual clades, rather than of multiple independent clades that encode similar functions. After producing these simulated profiles, we applied POMS, the phylogenetic regression workflows and the differential abundance approaches to identify significant KOs.

We evaluated the results based on the significance ranking of the focal gene relative to all other significant KOs identified by each method in a given replicate, such that the highest ranking (i.e. the rank of one) was assigned to the gene with the smallest *P*-value. Under our focal gene-based simulations, MAGs encoding the focal genes across the profiles were the only direct targets of selection, and accordingly the focal gene was expected to be highly ranked (i.e. close to one, indicating a smaller *P*-value compared to those of other genes).

**Fig. 2.**
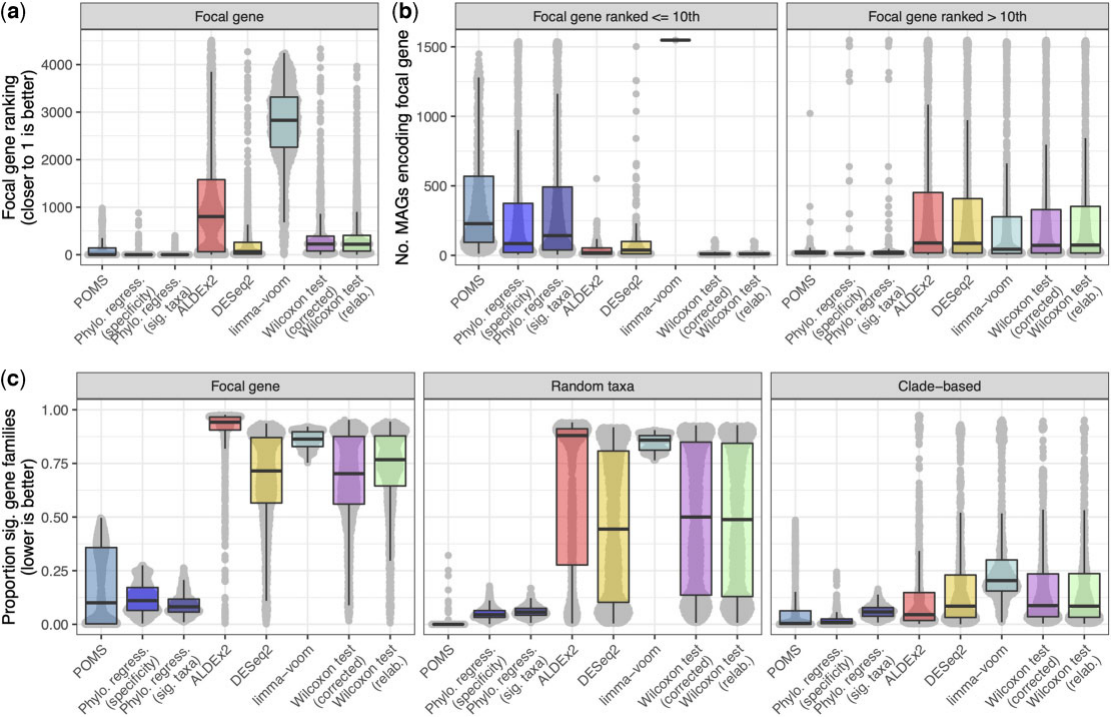
POMS performs best on simulated data based on focal gene rankings and the proportion of significant gene families. (**a**) Ranking of focal gene (i.e. the gene expected to be significant) in the output of all significant genes, based on *P*-values. No point is shown for cases where the focal gene was not significant. There was a total of 4710 gene families (KEGG orthologs) tested in each replicate. (**b**) Comparison of the numbers of MAGs that encode the focal gene in cases where the focal gene was ranked within the top 10 gene families in panel a versus cases where it was not. (**c**) Proportion of gene families identified as significantly different between the simulated sample groups by each approach. The ‘Focal gene’ panel represents the 1000 simulations where the MAGs that encode a specific gene family increased in abundance in one sample group. The ‘Random taxa’ panel corresponds to 1000 replicates where the abundance of randomly selected MAGs was perturbed in one sample group. Under these conditions no systematic differences in functional abundances would be expected on average. The ‘Clade-based’ panel represents the 693 instances where taxa underlying the same node were selected as the target of perturbation. In all panels, each gray point corresponds to one simulation replicate profile. Note that ‘lower is better’ is indicated on panel c to emphasize that identifying almost all functions as significant is not useful and that few genes are expected to be significant in the random taxa and clade-based tests

Comparing the approaches based on their distributions of focal gene rankings over the simulation replicates reveals drastic differences (Fig. 2a). The clearest difference is that the focal genes identified by all three phylogenetic methods are ranked significantly higher than were the focal genes identified by the differential abundance tools. The phylogenetic regression approaches ranked the focal genes the highest, with a median rank of one, followed by POMS with a median rank of two, and DESeq2 with a median rank of 65.75. We observed the same overall results based on simulations with less extreme selection pressures, and with altered number of MAGs (Supplementary Figs S2 and S3). In addition, we observed the same overall trend with simulated sparse abundance profiles based on reference genomes, which shows that this result is not driven by bias related to focusing on MAGs (Methods and Results; Supplementary Figs S4 and S5).

We next investigated what factors were underlying the variation in focal gene ranking across the simulation replicate profiles. We suspected that the number of MAGs that encoded each focal gene would markedly impact detection ability. Indeed, we found that focal genes were ranked highly overall based on the phylogenetic methods, except for those encoded by very few MAGs (Fig. 2b; Supplementary Fig. S6). For instance, focal genes identified by POMS with relative rankings within the top 10 ranked KOs were encoded by a median of 228 MAGs, whereas those not included in the top 10 ranked KOs were encoded by a median of only 16.5 MAGs. This trend was reversed for the differential abundance tests. For instance, focal genes in the DESeq2 output ranked within the top 10 KOs were encoded by a median of 38 MAGs, whereas those not included in the top 10 KOs were encoded by a median of 89 MAGs.

The approaches also varied in terms of how often they called the focal genes as significant. In particular, the focal gene was not called as significant by POMS in numerous cases: we found that the focal gene was significant in 78.8% of the POMS outputs, while it was called as significant in a mean of 95.1% of the outputs by all other tools. Most occurrences where the focal gene was not significant in the POMS output corresponded to cases where the gene was encoded by either extremely few or almost all MAGs (Supplementary Fig. S7). This result highlights the fundamental distinction between POMS and other approaches: POMS is more likely to identify significant genes that are consistently enriched in independent taxonomic lineages, which can be most clearly detected for widely encoded (but not ubiquitous) genes. In contrast, differential abundance—and to some extent phylogenetic regression—approaches call genes as significantly different even when these genes are linked to only a small number of taxa.

Having shown that the focal gene is successfully detected and highly ranked by POMS, we next turned to compare the overall number of significantly different KOs identified by POMS versus standard differential abundance tests. This analysis demonstrated that the proportion of significantly different KOs in the random taxa simulated profiles was clearly lowest based on POMS (mean = 0.001; SD = 0.0.017) compared to all other approaches [e.g. the next lowest was phylogenetic regression (specificity): mean = 0.051; SD = 0.028; Fig. 2c]. However, importantly, the random taxa and focal gene simulated profiles yielded substantially different results based on the phylogenetic methods, particularly for POMS, whereas the difference between the two profiles was less pronounced using the differential abundance approaches Specifically, there was a 12 026% increase in the mean proportion of significant KOs detected by POMS in the focal gene dataset compared to the random taxa dataset, while the corresponding increase based on DESeq2 (which had the highest such increase among the differential abundance tools) was 46.8%. In contrast, the mean proportion of significant KOs identified by POMS in the focal gene dataset was only 166% greater than the proportion identified in the clade-based analysis. These results suggest that POMS, and phylogenetic regression (Fig. 2c), better control the false positive rate in cases where no consistent functional differences are expected, but can still identify substantial number of significant hits when there is a true signal. Interestingly, the outliers in clade-based POMS results (Fig. 2c) indicate that this approach can erroneously call significant genes when only a single clade is shifted in abundance, at least in rare instances. This is clearly an unintended result and is important to appreciate (see Discussion). Nonetheless, a more striking concern is that the standard differential abundance tests frequently identified more than 50% of functions as significant even in cases where sample group differences were based on random taxonomic perturbations. These overall results were consistently observed regardless of parameter settings (Supplementary Fig. S8).

Finally, we also used modified versions of these simulated datasets (see Supplementary Methods) to assess the resource usage of the phylogenetic methods we implemented. We found that the POMS running time (on one core) increases linearly with the number of genomes (Supplementary Fig. S9). For instance, on one core the workflow took 69 s with 500 MAGs, and 130 s with 1000 MAGs. The phylogenetic regression workflows showed runtimes on the same order of magnitude, but ran slightly faster than POMS when the number of input MAGs was large. The regression workflows also consumed more than double the memory of POMS (max: 0.42 GB for POMS and 0.96 GB for the phylogenetic regression approaches).

### 3.3 Application to real datasets

We next applied all approaches discussed above to several real metagenomics datasets. Unlike in our simulated datasets, for these datasets we had no clear expectations regarding which functions would be identified as CEFs, and our evaluation is accordingly vulnerable to subjective biases in interpretation. Yet, as we argue below, many of the significant CEFs identified by POMS are reasonable given the presence of similar findings in the literature.

We first focused on MAGs assembled as part of the Tara Oceans project. Since strong selection pressures may act upon microbes across environmental gradients in the ocean (Ibarbalz *et al.*, 2019; Salazar *et al.*, 2019), these metagenomics samples represent an appealing test case for POMS. Our analysis focused specifically on 93 samples with 642 MAGs annotated with KOs. Environmental factors associated with the water samples were also available, including temperature, salinity, oxygen levels, nitrate concentrations, phosphate concentrations and nitrogen dioxide concentrations. We applied POMS to this dataset to detect microbial functions associated with each of these factors independently. We considered KEGG pathways and modules in addition to KOs for this analysis. These higher-level functions were computed for each MAG independently based on the KO annotations (see Section 2). Because the environmental factor data is continuous rather than partitioned into discrete groups, we identified BSNs based on Spearman correlations between the balances at each node and the factor levels. The direction of each BSN was inferred from the sign of the correlation coefficient. We also binned CEFs in this analysis as either stringent, intermediate or lenient, based on BH cutoffs of 0.05, 0.15 and 0.25, respectively.

Our analysis across all environmental factors identified in total one CEF below the intermediate cutoff and 19 CEFs below the lenient cutoff (Table 1). These CEFs were associated with either phosphate or mean salinity levels (for which there were 13 and 11 BSNs, respectively), and indeed some of the identified functions likely reflect selective actions of these two factors. For instance, a pathway linked to biofilm formation, ko05111, was found to be associated with higher phosphate levels, in agreement with previous findings of the non-trivial relationship between phosphorus levels and biofilm formation (Danhorn *et al.*, 2004; Fang *et al.*, 2009). Several significant CEFs linked to higher phosphate levels are also membrane and transport-related genes, which could be related to response to an environmental stress. Reasonable connections can also be made with the CEFs associated with mean salinity levels, such as the positive association with limonene and pinene degradation (ko00903). Limonene and pinene are monoterpenes that can be metabolized to modify cell membrane fluidity. This could reflect a potential adaptation that could facilitate an organism’s survival in the face of high salinity levels (De Carvalho and Fernandes, 2010).

**Table 1.** Tara oceans POMS results

Env. factor	Data type	Func. id.	Function description (simplified)	Total FSNs	FSNs ∩ BSNs (high)^a^	FSNs ∩ BSNs (low)^b^	FSNs not ∩ BSNs^c^	Corr. *P*	Sig. Reg.^d^
Phosphate	Pathway	ko05111	Biofilm formation—*V. cholerae*	13	8	0	5	0.13	No
Salinity	Pathway	ko00903	Limonene and pinene degradation	15	8	0	7	0.16	No
Phosphate	KO	K00705	4-Alpha glucanotransferase	9	7	2	0	0.17	No
Phosphate	KO	K01361	E3.4.21.96; lactocepin	7	6	1	0	0.17	Yes
Phosphate	KO	K01745	HAL; histidine ammonia-lyase	12	9	0	3	0.17	No
Phosphate	KO	K07137	Uncharacterized protein	12	9	1	2	0.17	Yes
Phosphate	KO	K15523	Protein-ribulosamine3-kinase	8	7	0	1	0.17	No
Phosphate	KO	K00151	5-Carboxymethyl-2-hydroxymuconic-semialdehyde dehydrogenase	12	8	0	4	0.20	No
Phosphate	KO	K01712	UROC1; urocanate hydratase	12	8	0	4	0.20	No
Phosphate	KO	K06872	Uncharacterized protein	12	8	0	4	0.20	No
Phosphate	KO	K06894	Alpha-2-macroglobulin	11	8	1	2	0.20	Yes
Phosphate	KO	K06940	Uncharacterized protein	12	8	0	4	0.20	No
Phosphate	KO	K07709	Two-component system, sensor histidine kinase HydH	5	5	0	0	0.20	No
Phosphate	KO	K08988	Putative membrane protein	9	7	0	2	0.20	No
Phosphate	KO	K09933	mtfA; MtfA peptidase	9	7	0	2	0.20	No
Phosphate	KO	K02065	Phospholipid/cholesterol/gamma-HCH transport system ATP-binding protein	17	9	0	8	0.24	Yes
Phosphate	KO	K15176	RNA polymerase-associated protein CTR9	6	5	1	0	0.24	No
Salinity	Module	M00040	Tyrosine biosynthesis	6	5	0	1	0.24	Yes
Salinity	Module	M00535	Isoleucine biosynthesis	10	6	0	4	0.24	No
Salinity	Module	M00879	Arginine succinyltransferase pathway	10	0	6	4	0.24	No

a FSNs intersecting BSNs associated with higher levels of environmental factor.

b FSNs intersecting BSNs associated with lower levels of environmental factor.

c FSNs not intersecting BSNs.

d Significant based on phylogenetic regression.

We next investigated how the results would differ if phylogenetic regression or Spearman correlations were used to test for associations (see Section 2). We found that using these approaches resulted in substantially more functions associated with each factor. For instance, there were 1008 and 1348 significant KOs (BH < 0.25) associated with mean salinity and phosphate levels, respectively, based on phylogenetic regression. Using Spearman correlation there were 4169 and 4974 significant KOs for mean salinity and phosphate levels, respectively. Only 10/20 and 5/20 CEFs identified by POMS were also significant in the Spearman correlation and phylogenetic regression outputs (Table 1), respectively. We also assessed the phylogenetic distribution of taxa encoding significant functions, based on Faith’s phylogenetic diversity. Taxa sets encoding POMS CEFs had slightly higher values (mean = 50.0; SD = 27.2), compared to phylogenetic regression hits (mean = 46.9; SD = 44.1), but the difference was not significant (Wilcoxon test, W = 18,123, *P* = 0.069; Supplementary Fig. S10a). In all cases, POMS-detected CEFs were not consistently amongst the most highly ranked hits by these other tools (Supplementary Fig. S11a).

We then investigated how POMS performs on case–control metagenomics datasets. We focused on a dataset of MAGs compiled from human-associated microbiomes that were published as part of a large-scale meta-analysis. We used subsets of this dataset corresponding to three disease datasets: two obesity datasets and one colorectal cancer dataset.

The first obesity dataset we analyzed included stool samples from 477 obese and 257 control individuals that collectively contained a total of 1401 MAGs. We applied POMS to this dataset and identified 34 BSNs that differed between obese and control individuals. The second obesity dataset analyzed included 251 obese and 159 control individuals harboring a collective total of 1161 MAGs in their stool microbiomes, with 31 resulting BSNs. POMS identified 79, 2 and 21 KOs, pathways and modules, respectively, which were consistently enriched in at least one dataset (Supplementary Table S1). Of these hits, two KOs, no pathways and two modules were called as CEFs across both datasets.

Importantly, many of these significant hits are reasonable given our knowledge of the link between the human microbiome and obesity. For example, one of the strongest CEFs detected by POMS was for the module cytochrome bd ubiquinol oxidase (M00153), which was associated with case samples. This function could reflect a broad shift in energy production through oxidative phosphorylation, or potential adaptation to oxidative stress, in obese individuals (Giuffrè *et al.*, 2014). Several other CEFs are also consistent with similar broad selection pressures due to dietary or metabolic differences. For instance, KOs involved in vitamin B1 and B2 biosynthesis (K00941 and K00794), as well as a module involved in vitamin K2 biosynthesis (M00116), were CEFs in case samples. The connection between these vitamins and obesity is controversial, but in certain cases they have been reported at lower levels in obese individuals (Gunanti *et al.*, 2014; Ravera *et al.*, 2021). Similarly, beta-lactam resistance (ko01501) was also significantly associated with case samples, which is particularly interesting as exposure to antibiotics has long been known to be associated with obesity (Wilkins and Reimer, 2021). Thus, one hypothesis consistent with these results is that these functions represent widespread adaptations to stresses and the resource landscape in the gut environment of obese individuals.

Last, we applied POMS to the microbial profiles of stool samples from 75 colorectal cancer patients and 53 controls, which contained 1187 MAGs. In this case, there were only 14 BSNs and no significant CEFs. Nonetheless, the sole outlier in the results (the only feature with corrected *P* < 0.6) was glyoxylate and dicarboxylate metabolism (enriched in control patients; corrected *P* = 0.294). This outlier is noteworthy, as it has previously been identified as the most significantly depleted function in colorectal cancer samples (relative to controls) based on metabolite profiles (Arima *et al.*, 2020).

In contrast to the POMS results, phylogenetic regression (specificity-based) resulted in many more hits in the colorectal cancer dataset (2996) than in the obesity dataset (1202). In addition, no pathways, and only 14/79 KOs and 4/21 modules (Supplementary Table S1) were called as significant by both approaches in the same dataset. In this case, taxa sets encoding POMS CEFs exhibited significantly higher values of Faith’s phylogenetic diversity (mean = 114.9; SD = 69.9; Wilcoxon test, W = 80,348; *P* = 4.0 × 10^−7^), compared to those encoding the phylogenetic regression hits (mean = 80.2; SD = 74.0; Supplementary Fig. S10b). In addition, POMS CEFs were not amongst the highest ranked significant calls based on phylogenetic regression or the differential abundance approaches (Supplementary Fig. S11b–d). These results highlight that POMS identifies distinct sets of significant hits compared with both other phylogenetic methods and standard differential abundance tests.

## 4 Discussion

Herein we have presented and validated the POMS framework: a novel approach for identifying CEFs in microbiome data. Using simulations, we have demonstrated that POMS can accurately identify widely encoded microbial functions that confer a strong selective advantage. While we present several different analyses to validate and justify our approach, perhaps the most convincing evidence is that focal genes (i.e. genes that were simulated as conferring a selective advantage) were amongst the most significant functions identified by POMS, whereas this was often not the case for standard differential abundance approaches. On the other hand, the focal genes were frequently identified by phylogenetic regression as well, but it is much more difficult to interpret the output of this approach. In addition, although POMS produced sensible results on real shotgun metagenomics datasets that were assembled into MAGs, these CEFs were typically not top hits based on phylogenetic regression. This suggests that POMS and phylogenetic regression are complementary tools that potentially give insight into different types of functional associations.

For instance, POMS can only identify functions that are widely encoded by taxa and that are variably present across different lineages; a gene restricted to an individual lineage, even if that lineage has many members, should not be identified as a CEF (although this can occur: see discussion of clade-based simulations below). This is because POMS requires a function to be enriched repeatedly (and in consistent directions) at independent BSNs to identify a function as significant. These functions necessarily will show a signal at deeper nodes in the tree, rather than on small clades or tips. This difference is reflected in the fact that taxa encoding significant POMS hits often had significantly higher Faith’s phylogenetic diversity compared to those encoding significant phylogenetic regressions hits. Consequently, POMS may miss many functions that would be identified by phylogenetic regression, and which may exhibit signal at sporadic tips in the tree. However, CEFs identified by POMS have a clear biological interpretation: each CEF represents the hypothesis that the function provides a fitness advantage in certain contexts to those taxa which encode it. This need not be the case for significant hits identified by phylogenetic regression: a small number of taxa may simply have bloomed or become depleted for a different reason.

It should also be appreciated that significant CEFs identified by POMS are not guaranteed to be enriched only in a single direction. The POMS multinomial test evaluates whether the distribution of FSNs into the three categories departs from the random expectation. Significant functions could simply be enriched at BSNs of both types, i.e. a mixture of BSNs relatively higher in both sample groups. Such cases could still be biologically interesting but would be interpreted differently than CEFs primarily enriched toward a single sample group. Accordingly, the counts of FSNs of each category should be considered when interpreting any CEFs identified by POMS.

There are also other limitations to the POMS approach. For instance, identifying CEFs requires that sufficient BSNs are present to identify significant enrichments. Even if an adaptive function is widely distributed phylogenetically, it will not be identifiable by POMS unless there are corresponding BSNs that could be driven by this function. In addition, POMS assumes that all nodes in the tree have independent balance distributions. This is partially invalid because particular taxonomic groups are more likely to vary across individuals and taxon co-occurrence can occur even at long evolutionary distances (Ma *et al.*, 2020). It is also a caveat for all balance tree approaches that there can be correlations in node balances simply due to the same tips underlying the nodes. We assessed the extent of this problem with our clade-based perturbation simulations, which indicated that on occasion POMS can produce erroneous results due to this issue. However, the proportion of significant KOs was still much lower than for the differential abundance tools. Future development that focused on building a similar framework based on phylofactorization (Washburne *et al.*, 2019) instead of balance trees could help address this issue.

Despite these caveats, we have shown that compared with more common approaches, integrating functional information into a balance tree framework can better identify functions that could provide a selective advantage. POMS is just one example of how this general analysis scheme can be implemented, and future work could incorporate more sophisticated approaches for analyzing balances across phylogenetic trees (Silverman *et al.*, 2017; Washburne *et al.*, 2019). Nonetheless, the current POMS framework represents a more robust methodology compared to the common practice of applying differential abundance tests to community-wide functional abundances. More generally, given the wide range of results yielded by popular microbiome differential abundance approaches (Nearing *et al.*, 2022) and their poor performance in our simulation experiments, clearly phylogenetic methods should be preferred in this context.

## Supplementary Material

btac655_Supplementary_Data

## Data Availability

The Tara Oceans MAG dataset was acquired from FigShare (10.6084/m9.figshare.4902923.v1). The human case–control datasets are available on the NCBI short read archive under accessions ERP002061 (obesity #1), ERP003612 (obesity #2) and ERP012177 (colorectal cancer). Our analyses were based on the MAGs and related data files that were made available by a meta-analysis of these and other studies (Almeida *et al.*, 2019). The prepared in-files for running the simulations and actual data examples we describe, as well as the output tables of the phylogenetic methods applied to the actual datasets, are available on FigShare at 10.6084/m9.figshare.c.6162060.v2.
