# Supplementary material for: Integrating phylogenetic and functional data in microbiome studies
Source: Bioinformatics. 2022 Sep 30;38(22):5055–63. doi: 10.1093/bioinformatics/btac655 (PMC9665866; doi:10.1093/bioinformatics/btac655)
Supplement: btac655_Supplementary_Data [file btac655_supplementary_data.pdf]

Supplementary Methods and Results

Exploring the parameter space of the MAG-based simulations

The selection simulation framework that we describe in the main text represents an extremely strong selection acting upon taxa encoding the focal gene in each profile. Specifically, this simulated selective advantage included (in the sample group experiencing selection) adding a pseudocount of one to the abundance of all taxa that encoded the focal gene and then multiplying the resulting abundance by 1.5.

To confirm that POMS is not only performing better under this extreme condition, we also created and tested simulated profiles over a range of weaker selection settings. These altered settings included not adding a pseudocount and/or multiplying the abundances by lower factors (1.05 and 1.25, in addition to the original value of 1.5). In addition to either adding a pseudocount or not, we also varied what proportion of relevant metagenome-assembled genomes (MAGs) would receive a pseudocount (the pseudocount itself was 1 in all cases), testing specifically proportions of 0, 0.3, 0.7, and 1. These proportions represent the proportion of randomly sampled MAGs, of the subset that encode the focal gene, whose abundance is increased by one. We additionally tested the impact of different numbers of MAGs in these simulations, to investigate how dataset size affects the POMS framework. We varied the number of MAGs between these values: 100, 250, 500, 1000, and 1595 MAGs. To keep this analysis manageable, we considered only 10 independent replicates for each of the 60 combinations of these simulation settings. To enable clearer comparisons between replicates with differing number of MAGs, we considered the same 10 KEGG orthologs to be the focal genes for all settings combinations. When subsampling MAGs, this was done randomly except that the proportion of MAGs encoding the focal KEGG ortholog was kept as similar as possible (to a minimum of five MAGs). This analysis clearly demonstrated that across most settings the focal genes were substantially more highly ranked by the phylogenetic methods compared with the Wilcoxon test approach (**Supp. Figure 2**). The Wilcoxon test was chosen as the representative differential abundance test for these additional experiments due to its high speed of computation. The same overall trends for the random taxa and clade-based analyses presented in the main text were also observed consistently under these varied parameter settings.

The exception to the above observation was in simulation settings where no pseudocount was added, in which the focal gene was largely non-significant in the POMS output due to insufficient statistical power. This drop in statistical power is reflected by lower numbers of balance-significant nodes (BSNs) on average for these settings (**Supp. Figure 3**). In contrast, the focal genes were highly ranked on average based on the Wilcoxon test for these simulation settings.

Our analysis also demonstrated a marked drop in the number of BSNs in simulations with 250 or fewer MAGs (**Supp. Figure 3**). POMS was unable to call any focal genes as significant in these simulations due to the small number of BSNs, while many were called as significant by other approaches.

Finally, these analyses also demonstrated that two other observations reported in the main text—the overall tendency of phylogenetic regression to result in a non-negligible proportion of significant hits under the random taxa and clade-based simulations, as well as the observation that the Wilcoxon test identifies a higher proportion of significant hits overall—were robust to the simulation settings (**Supp. Figure 8**).

Reference-genome-based simulations

Our observations based on the MAG-based simulations are valuable, but one caveat is that the quality of published MAGs is often questionable[1]. To ensure that misassembled MAGs were not driving our results, we repeated our simulation approach on 500 reference genomes.

These genomes were taken from the Integrated Microbial Genomes database[2] and were previously parsed for use with PICRUSt2[3]. Per-genome KEGG ortholog annotations were taken from the default PICRUSt2 database. To clarify, these annotations are based on complete genomes (i.e., with no imputation) and were obtained from the PICRUSt2 database as it provided a convenient and easy-to-use format for our analyses. We created a de novo phylogenetic tree based on a set of universal single-copy genes with GToTree[4] v1.4.16. This approach parses out universal single-copy genes from genome sequences and wraps several tools to build a phylogenetic tree. The tool was run with the bacterial hidden Markov model setting and with FastTree[5] v2.1.10. GToTree also returns estimates of the percent completeness and redundancy for each genome. We excluded all genomes with completeness below 95% and/or redundancy

above 5%. We then randomly sampled 500 of the remaining high-quality genomes for the subsequent analyses.

We next simulated random abundances of these genomes across 1000 samples based on the zero-inflated beta distribution implemented in the rBEZI function of the gamlss.dist v5.1.7 R package[6]. Simulations under this model can be modified with three key metrics: mu (the mean), nu (the probability of zero abundance), and sigma (the standard deviation). For these simulations we maintained values of mu and sigma of 0.1 and 1, respectively, throughout. We generated four simulated datasets based on nu values set to 0.5, 0.65, 0.8, and 0.95. When generating these simulated datasets we required that each sample contain a minimum of five genomes (i.e., simulated profiles were re-run if fewer than five genomes had non-zero abundance). These altered nu values had a large impact on the sparsity and inter-sample overlap of each dataset (**Supp. Figure 4a**).

We then re-ran the key steps of our simulation analysis on these simulated abundance tables and genomes. Specifically, we repeated the focal gene-based simulation, including applying POMS, both phylogenetic regression approaches, and the Wilcoxon tests (USCG-corrected), over 500 replicate profiles for each of the four datasets. In all cases, we filtered out genes found in <5 genomes prior to running analyses. The Wilcoxon test was applied after taking the cross-product of the taxonomic abundances and taxa gene copy numbers to get the sample-wise gene abundances, as in the main text. The focal gene ranks varied substantially depending on the abundance table simulation approach (**Supp. Figure 4b**). The focal genes were ranked significantly highest for the Wilcoxon approach under the low sparsity settings (the distributions were significantly different [Wilcoxon test $P < 0.01$] in all cases). For example, when nu = 0.5, the median gene ranks were one and 13.5 for the Wilcoxon test and POMS, respectively. This trend reversed with the sparser abundance tables with nu = 0.8 (POMS median rank: 16; Wilcoxon test median rank: 19.5) and nu = 0.95 (POMS median rank: 34.25; Wilcoxon test median rank: 968). Notably, sparse abundance tables are the norm in microbiome data, and the non-sparse settings indicated in **Supp Figure 4a** are biologically unrealistic in virtually all environments.

We also investigated the relationship between focal gene ranks and the number of genomes encoding the focal gene. Like in the MAG-based simulation results, those significant focal genes in the POMS output that were not amongst the most significant genes (i.e., focal

genes at higher ranks) were encoded by fewer genomes (**Supp. Figure 5**). This was true for the other tested approaches as well, overall. However, the focal gene ranks based on Wilcoxon test p-values displayed a positive linear relationship with the number of encoding genomes for the sparsest simulated dataset (nu = 0.95). There was a significant positive correlation in this case (Pearson R = 0.756; $P < 10^{-15}$). This result was similar to the positive correlation between focal gene rank and number of encoding genomes that we also observed in the corresponding analysis on the MAG-based simulations (**Supp. Figure 6**). Overall, these reference genome-based simulation results are consistent with the key observations from the MAG-based simulations, at least when taxa are sparsely distributed, which is characteristic of microbiome datasets.

References

1. Shaiber, A. & Eren, A. M. Composite metagenome-assembled genomes reduce the Quality of public genome repositories. *MBio* **10**, e00725 (2019).
2. Markowitz, V. M. *et al.* IMG: The integrated microbial genomes database and comparative analysis system. *Nucleic Acids Res.* **40**, 115–122 (2012).
3. Douglas, G. M. *et al.* PICRUSt2 for prediction of metagenome functions. *Nat. Biotechnol.* **38**, 685–688 (2020).
4. Lee, M. D. & Ponty, Y. GToTree: A user-friendly workflow for phylogenomics. *Bioinformatics* **35**, 4162–4164 (2019).
5. Price, M. N., Dehal, P. S. & Arkin, A. P. FastTree 2 - Approximately maximum-likelihood trees for large alignments. *PLOS One* **5**, e9490 (2010).
6. Stasinopoulos, M. & Rigby, R. gamlss.dist: Distributions for generalized additive models for location scale and shape. (2020).

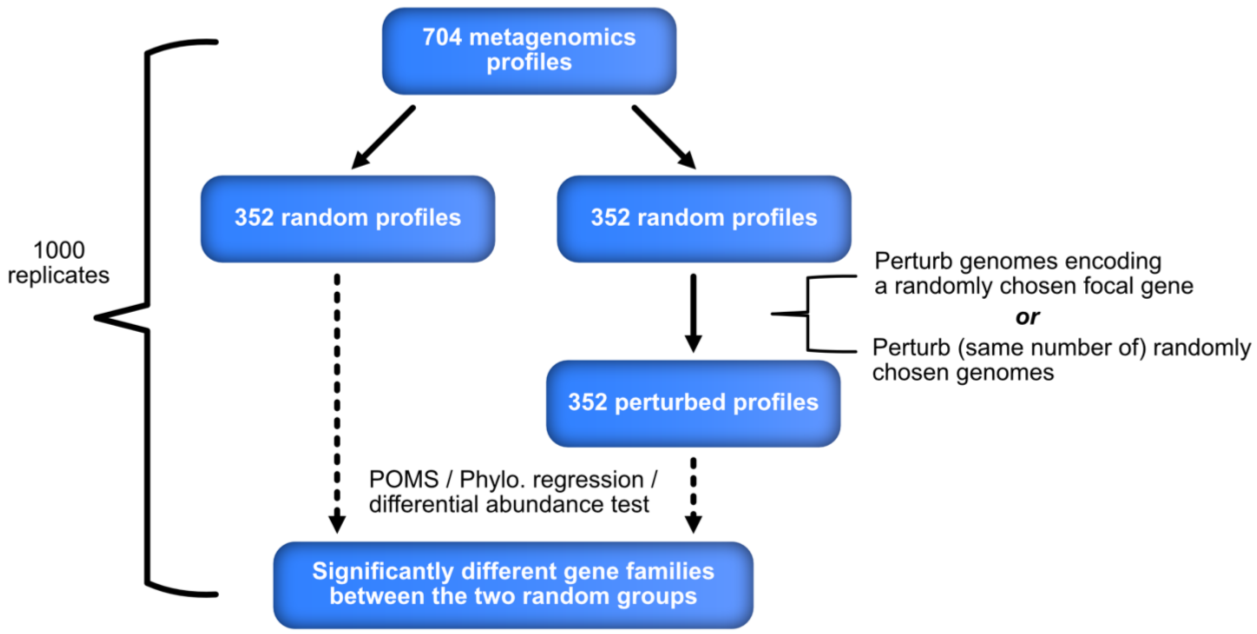

Supplementary Figure 1: Workflow diagram for metagenome-assembled genome-based simulations.

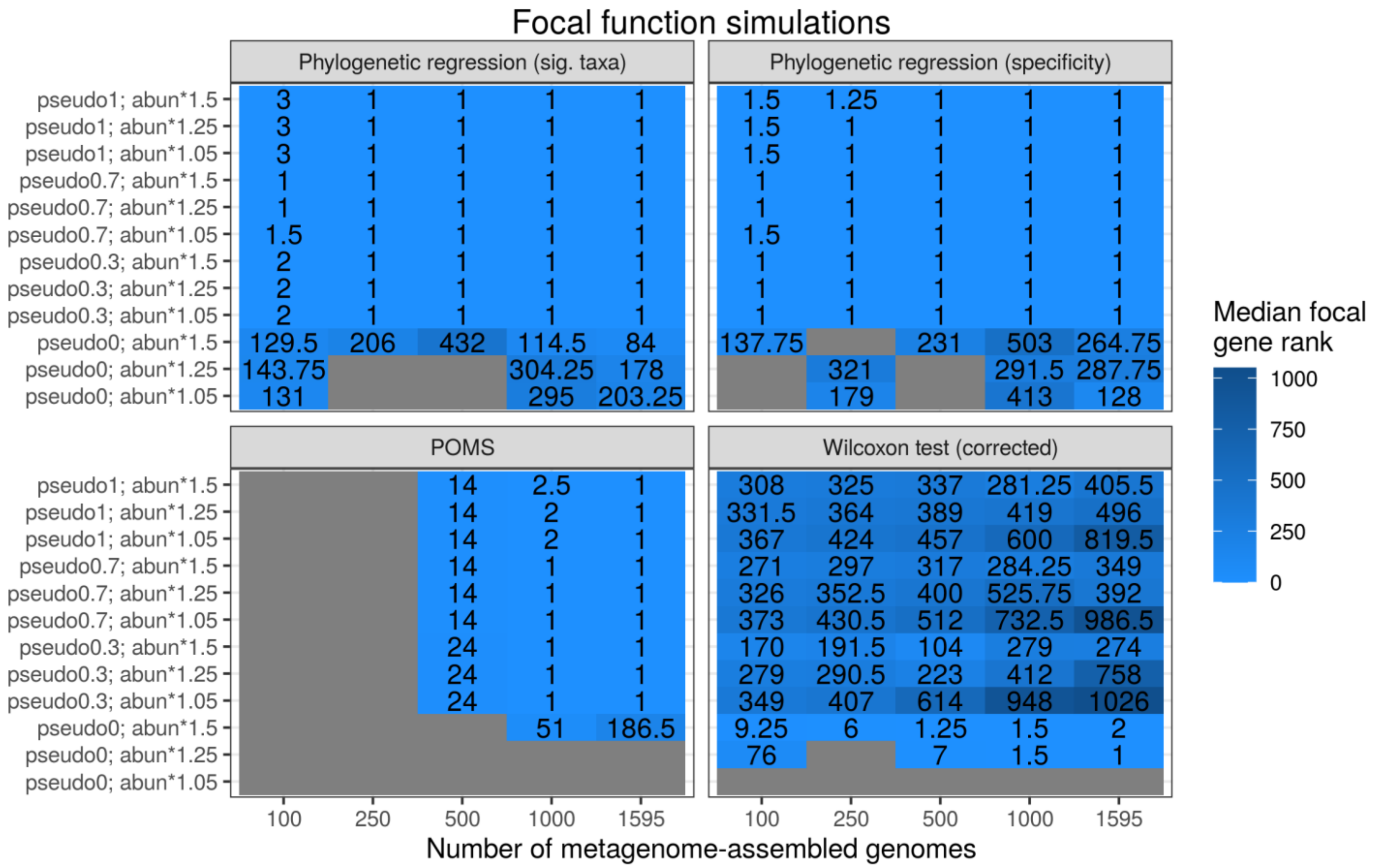

Supplementary Figure 2: Median significance ranking of focal gene determined by representative tools across replicates per simulation setting. The "pseudo" setting is the proportion of metagenome-assembled genomes (MAGs) that encoded the focal gene that were randomly selected per sample to have a pseudocount of 1 added to their abundance. The "abun" setting represents the scaling factor of the abundance of each genome encoding the focal gene after addition of the pseudocount. Grey boxes indicate cases where the focal gene was not significant in any replicate. There were 4710 genes tested as part of the full dataset (with 1595 MAGs). For each dataset of decreasing size there are fewer genes tested. On average there were 2342.3, 3040.9, 3786.0, and 4419.7 for datasets of size 100, 250, 500, and 1000 MAGs, respectively.

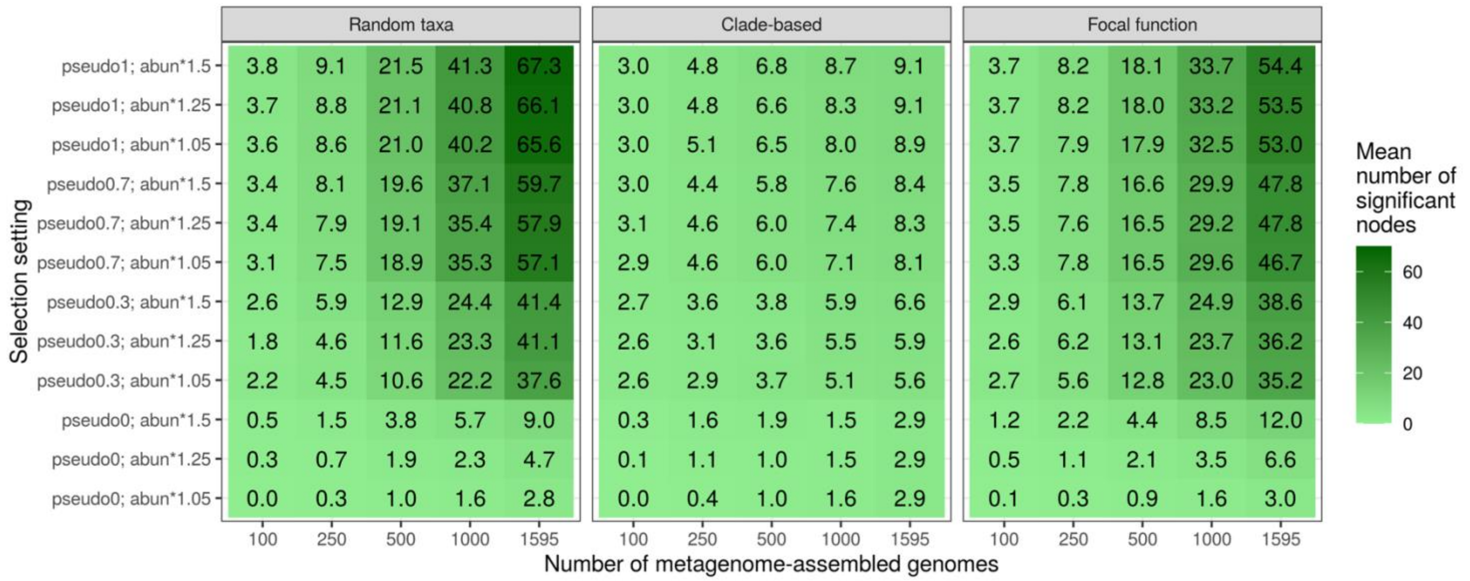

Supplementary Figure 3: Mean number of significant nodes based on sample balances (i.e., balance-significant nodes) across replicates for each simulation setting. The “pseudo” setting is the proportion of metagenome-assembled genomes that encoded the focal gene that were randomly selected per sample to have a pseudocount of 1 added to their abundance. The “abun” setting represents the scaling factor of the abundance of each genome encoding the focal gene after this pseudocount step.

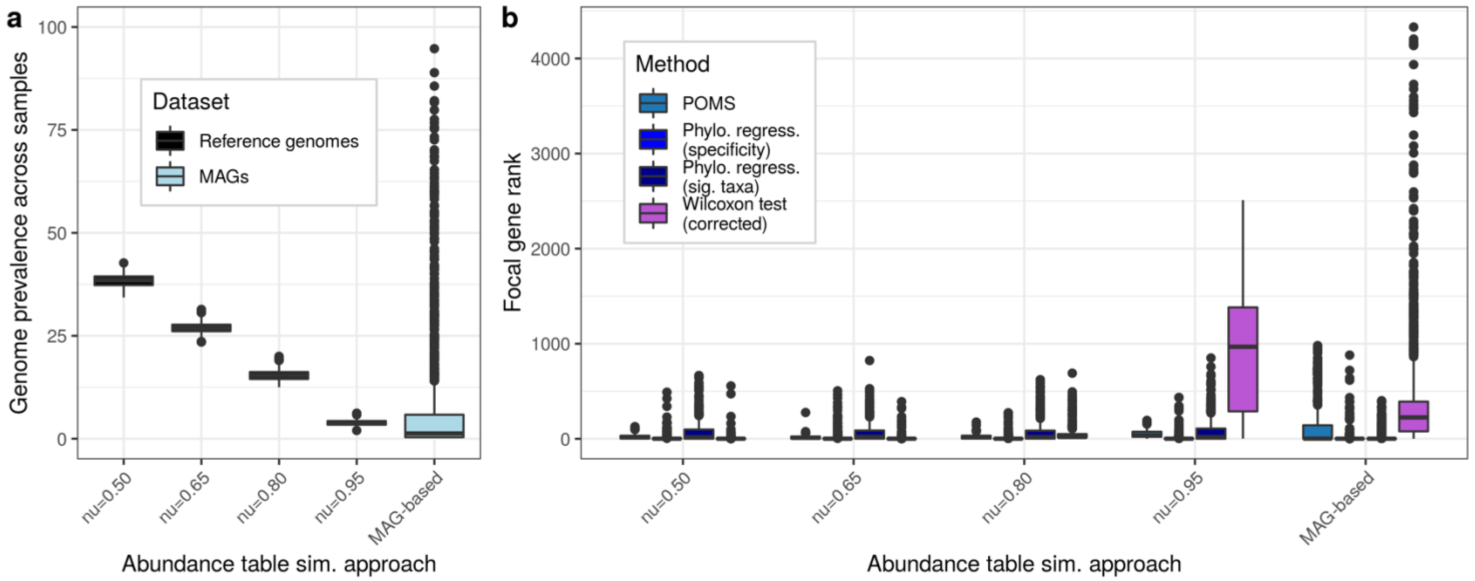

Supplementary Figure 4: Genome prevalence and ranking percentiles of focal genes varies across all simulation datasets. (a) The prevalence (%) of each genome (or metagenome-assembled genome [MAG]) across all samples in a dataset. (b) The ranking percentiles of the focal gene within the list of significant genes for each simulated dataset setting. The reference genome-based simulated datasets were altered based on four parameter settings, which greatly affect genome prevalence. The “MAG-based” group corresponds to the simulation results shown in the main text, which were included to enable comparisons with those results. There were 6191 genes tested for all reference-genome based simulations, and 4710 genes tested as part of the MAG-based validations.

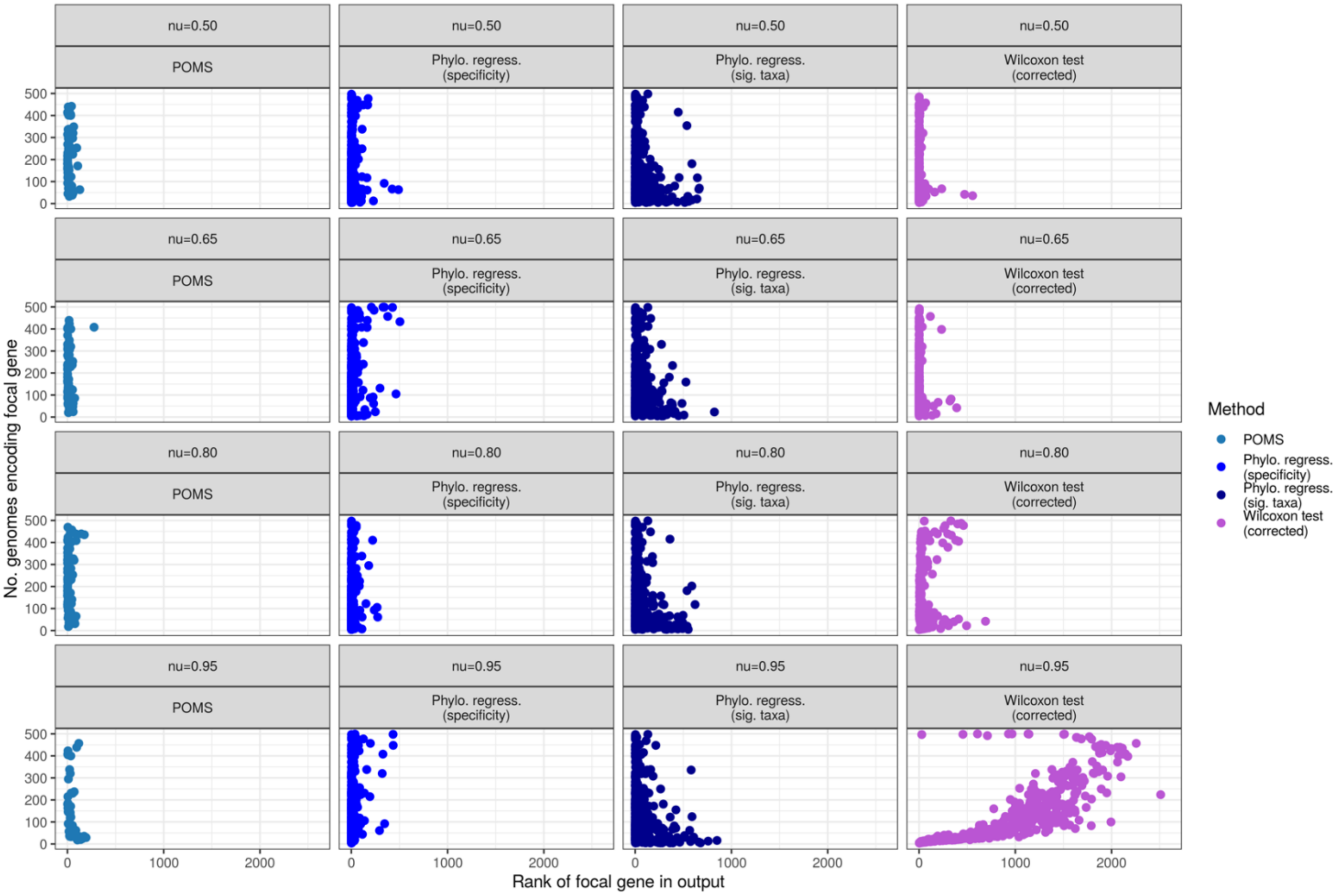

Supplementary Figure 5: Focal gene ranks versus the number of genomes in which they are encoded, based on reference genome simulations. Simulation setting (determined by the value of nu, the probability that a feature has an abundance of 0), and tool name, are indicated above each panel. Each panel matches a boxplot shown in Supplementary Figure 4b. There were 6191 genes tested for all reference-genome based simulations.

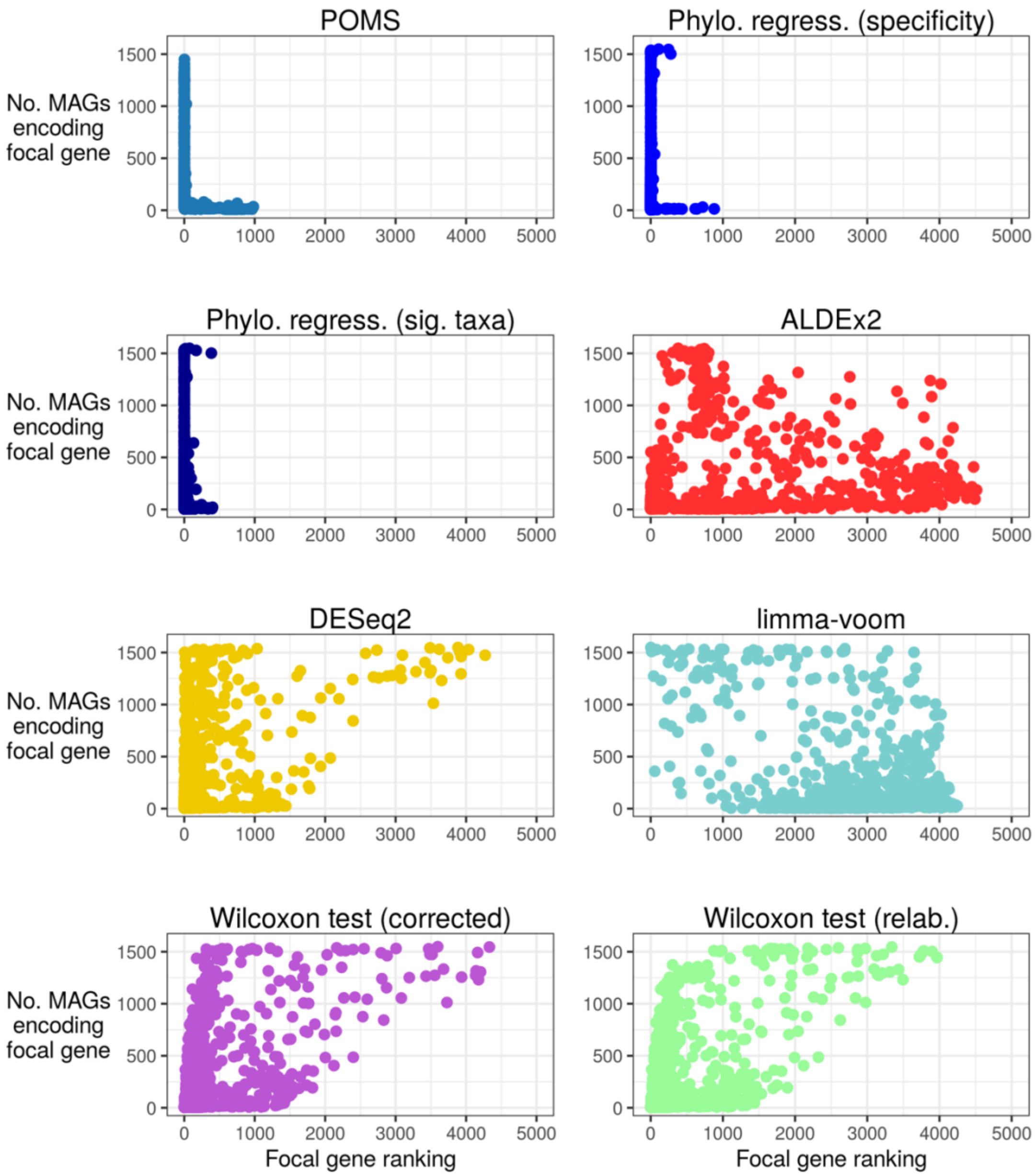

Supplementary Figure 6: Focal gene rankings are strongly associated with the number of metagenome-assembled genomes (MAGs) in which the focal gene is encoded. Each point is a simulation replicate analyzed with each of the approaches described in the main text. Each scatterplot represents the distribution of how the focal gene was ranked (1 being most significant) in the output of each tool, against how many MAGs encoded that gene. Only replicates where the focal gene was significant are shown. There were 4710 genes (KEGG orthologs) tested in all cases.

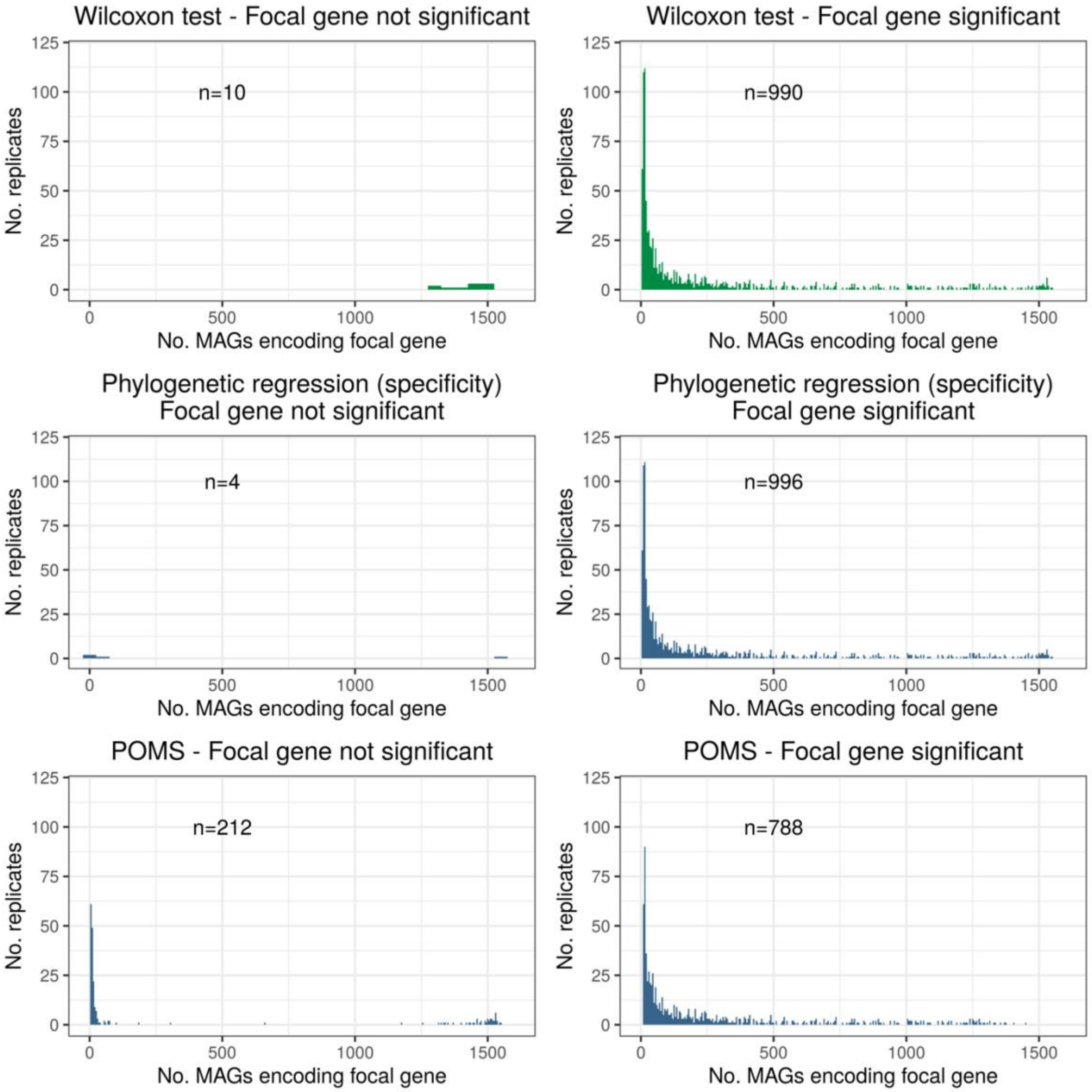

Supplementary Figure 7: Number of metagenome-assembled genomes (MAGs) that encode the focal gene in cases where it was called as significant and cases where it was not. The tested approach and result category are indicated above each panel. Bin sizes of 5 were used for all panels, except for non-significant phylogenetic regression and Wilcoxon test panels, where a bin size of 50 was used to allow improved visualization. The number of replicates is indicated in each panel.

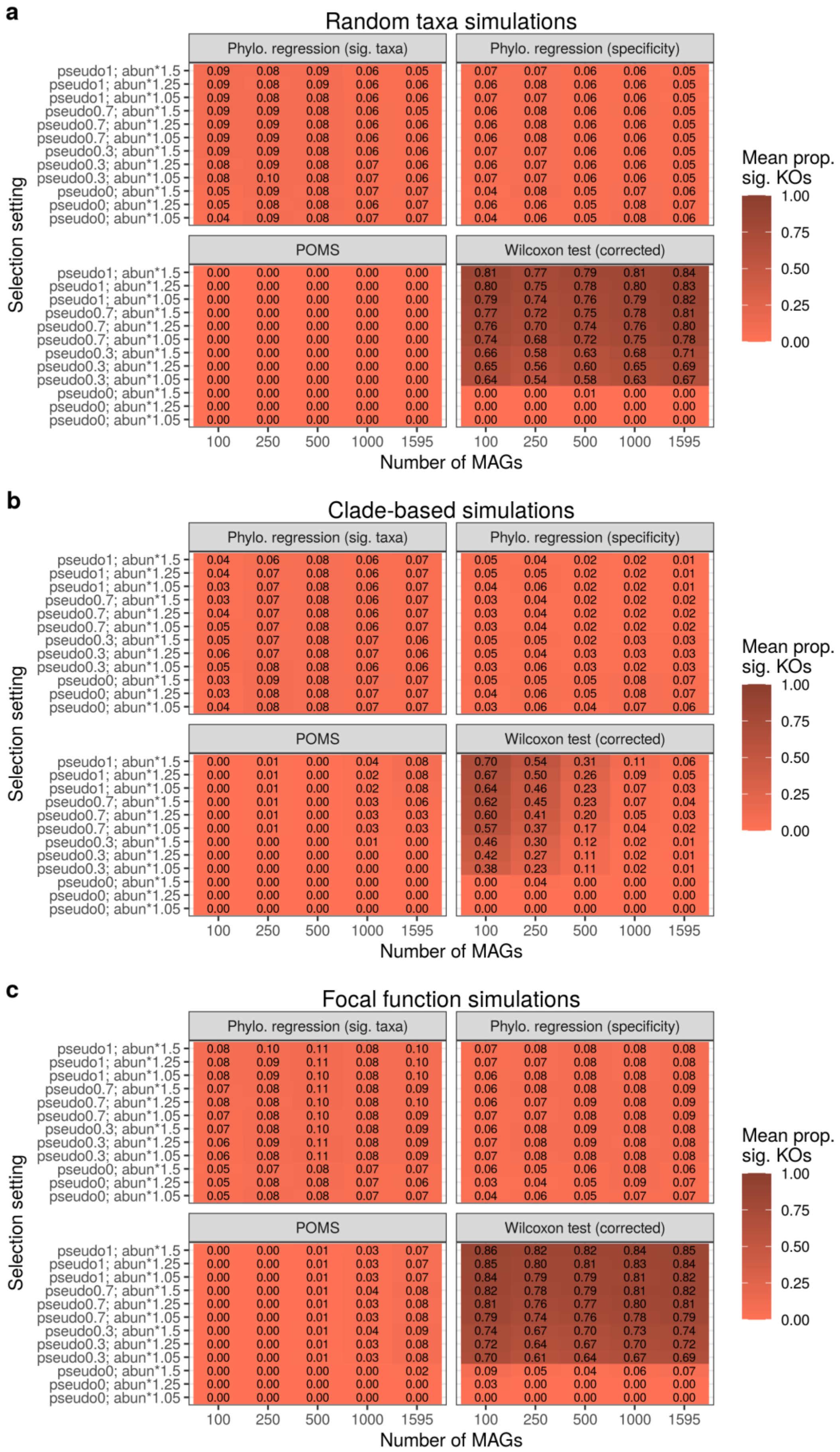

Supplementary Figure 8: Mean proportion of significant functions in output of the two phylogenetic regression workflows, the Wilcoxon test, and POMS across focal gene-based replicates per simulation setting. The three panels (a-c) correspond to the three simulation approaches described in the main text. The “pseudo” setting is the proportion of metagenome-assembled genomes (MAGs) that encoded the focal gene that were randomly selected per sample to be given a pseudocount of 1 to their abundance. The “abun” setting represents the scaling factor of the abundance of each genome encoding the focal gene after this pseudocount step. KOs: KEGG orthologs (i.e., the tested functions).

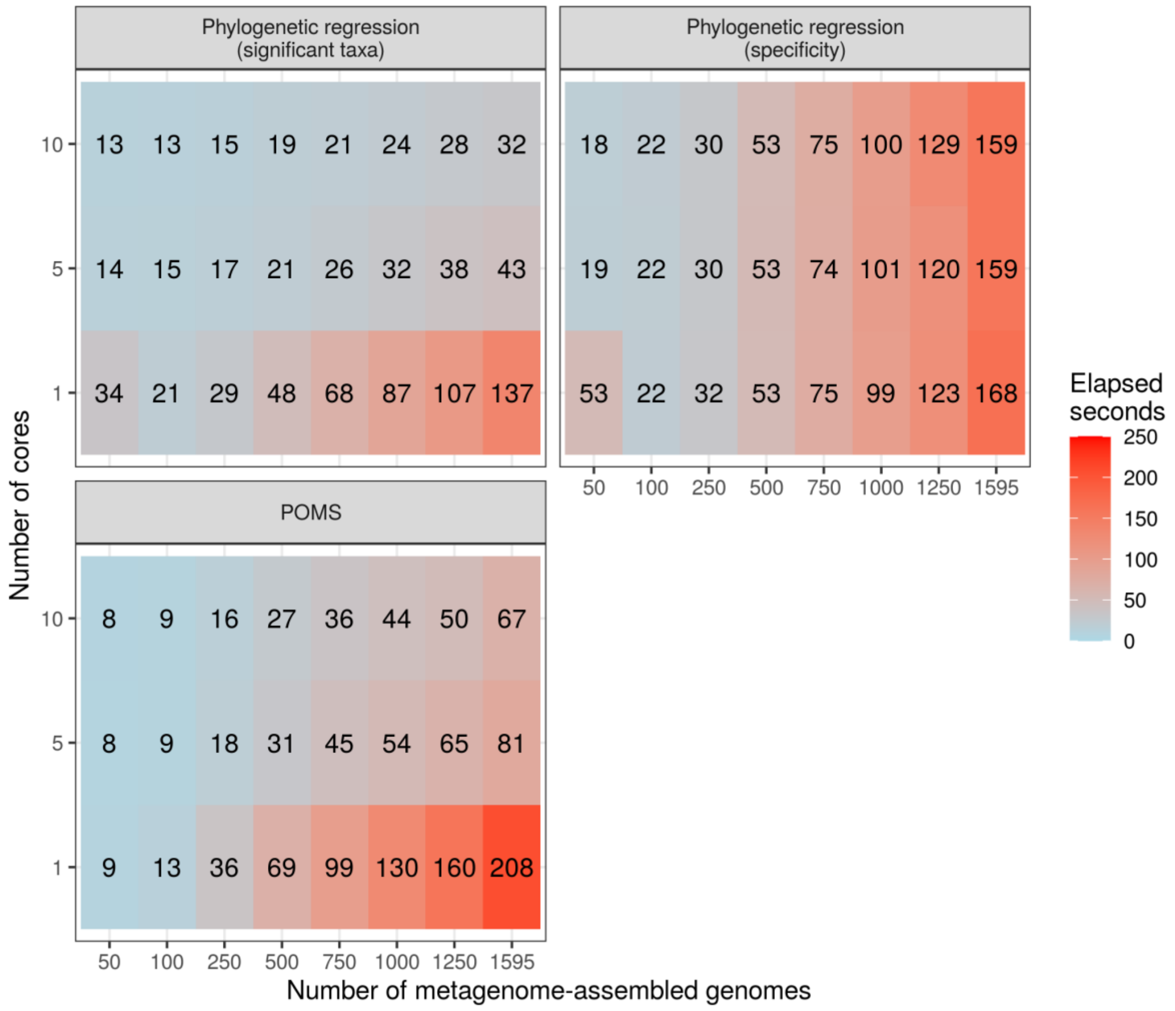

Supplementary Figure 9: Runtime for phylogenetic regression approaches and POMS. Each runtime shown is a mean of three replicates. Timing includes entire workflow (e.g., the time to read in the in-files and compute the specificity scores is included for the upper-right panel).

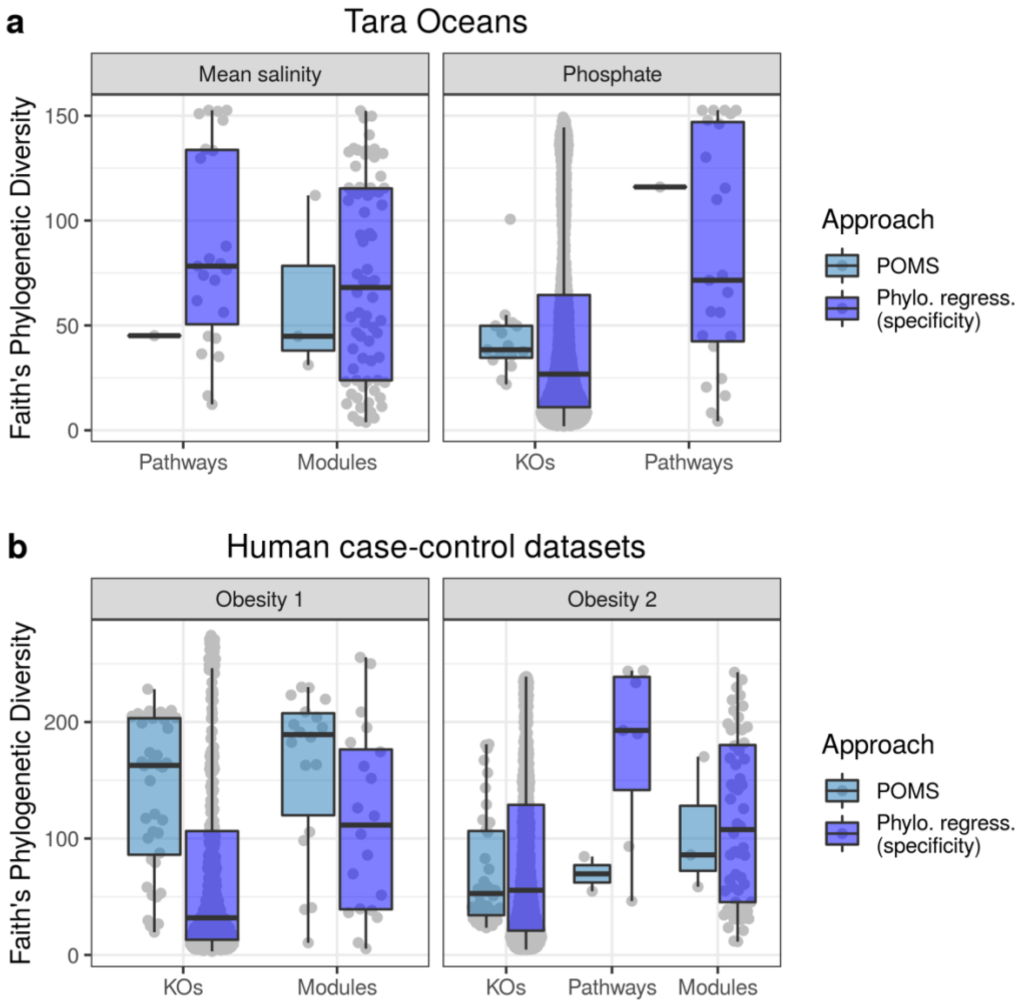

Supplementary Figure 10: Faith's phylogenetic diversity of taxa that encode significant functions in (a) Tara Oceans and (b) human case-control datasets. Each point is a function that was identified as significant (false discovery rate < 0.25) based on POMS or phylogenetic regression. Faith's phylogenetic distance is the sum of all branch lengths connecting all taxa in a phylogenetic tree (restricted to taxa that encode a given significant function in this case). Data types (e.g., KOs and pathways) without significant hits output by both approaches were dropped. KOs: KEGG orthologs.

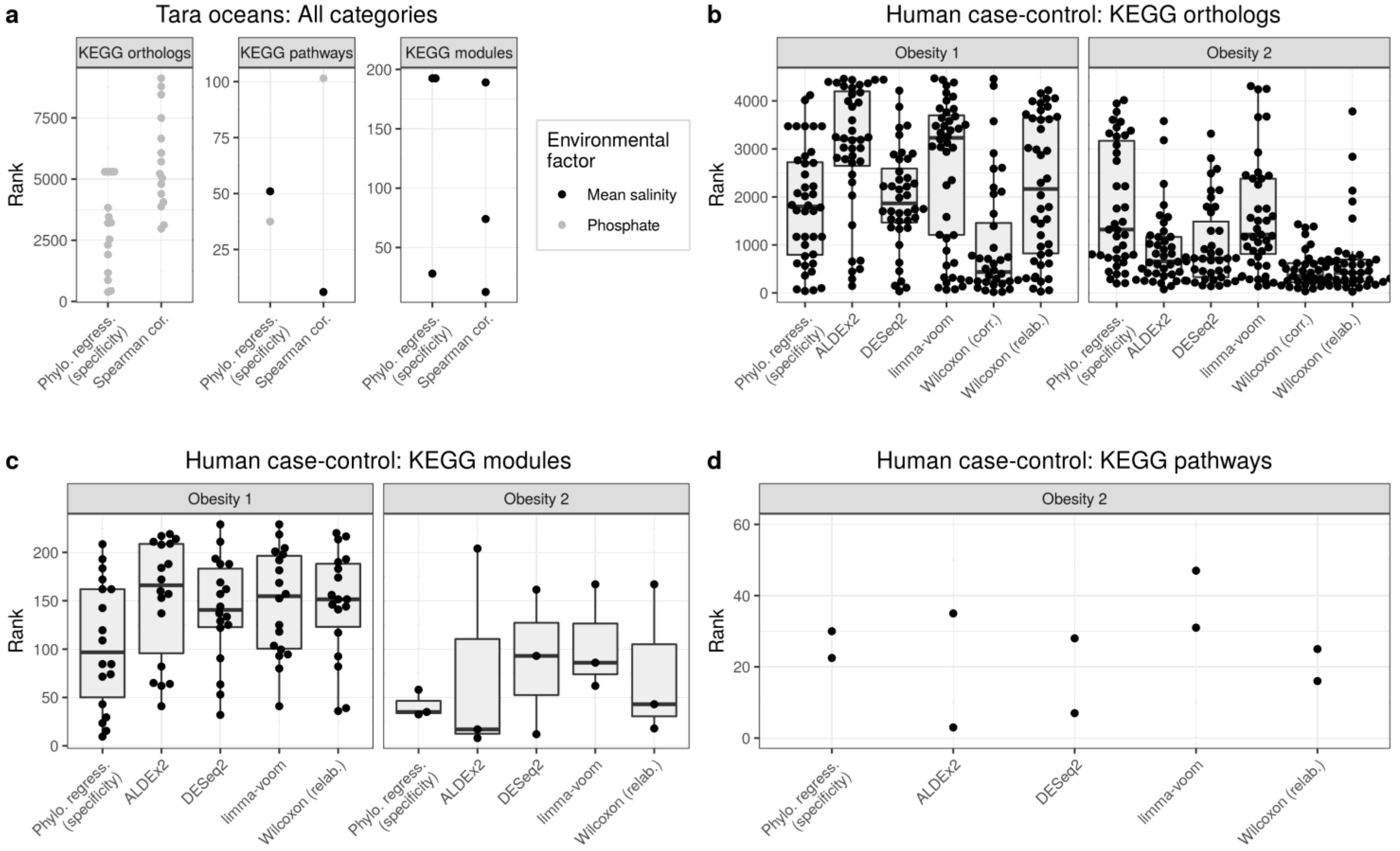

Supplementary Figure 11: Consistently enriched functions (CEFs) in POMS output are not generally amongst the top significant hits based on other tested approaches. Each dot corresponds to the rank of a CEF (i.e., a function identified as significant based on POMS) in the list of significant functions identified by an alternative tool.

Supplementary Table 1: Significant KEGG pathways and modules in obesity datasets based on POMS (KEGG orthologs were excluded here due to the high number of hits)

Dataset	Data type	Func. id.	Function description	Total FSNs	FSNs ∩ obese-associated BSNs	FSNs ∩ control-associated BSNs	FSNs not ∩ BSNs	Corr. P	Sig. Reg.[a]
Obesity 2	Module	M00116	Menaquinone biosynthesis, chorismate (+ polyprenyl-PP) => menaquinol	16	10	0	6	0.07	Yes
Obesity 2	Module	M00153	Cytochrome bd ubiquinol oxidase	13	10	1	2	0.07	Yes
Obesity 2	Pathway	ko00540	Lipopolysaccharide biosynthesis	15	9	0	6	0.09	No
Obesity 2	Pathway	ko01501	beta-Lactam resistance	16	8	0	8	0.15	No
Obesity 2	Module	M00082	Fatty acid biosynthesis, initiation	22	13	1	8	0.17	No
Obesity 1	Module	M00048	Inosine monophosphate biosynthesis, PRPP + glutamine => IMP	18	10	0	8	0.18	No
Obesity 1	Module	M00008	Entner-Doudoroff pathway, glucose-6P => glyceraldehyde-3P + pyruvate	26	11	1	14	0.21	No
Obesity 1	Module	M00015	Proline biosynthesis, glutamate => proline	23	11	1	11	0.21	No
Obesity 1	Module	M00051	Uridine monophosphate biosynthesis, glutamine (+ PRPP) => UMP	21	11	1	9	0.21	No
Obesity 1	Module	M00075	N-glycan biosynthesis, complex type	7	0	5	2	0.21	No
Obesity 1	Module	M00082	Fatty acid biosynthesis, initiation	25	12	2	11	0.21	Yes
Obesity 1	Module	M00116	Menaquinone biosynthesis, chorismate (+ polyprenyl-PP) => menaquinol	22	11	1	10	0.21	No
Obesity 1	Module	M00525	Lysine biosynthesis, acetyl-DAP pathway, aspartate => lysine	24	11	1	12	0.21	No
Obesity 1	Module	M00526	Lysine biosynthesis, DAP dehydrogenase pathway, aspartate => lysine	25	13	1	11	0.21	No
Obesity 1	Module	M00535	Isoleucine biosynthesis, pyruvate => 2-oxobutanoate	34	13	2	19	0.21	No
Obesity 1	Module	M00844	Arginine biosynthesis, ornithine => arginine	25	13	2	10	0.21	No
Obesity 1	Module	M00880	Molybdenum cofactor biosynthesis, GTP => molybdenum cofactor	27	14	5	8	0.21	No
Obesity 1	Module	M00845	Arginine biosynthesis, glutamate => acetylcitrulline => arginine	26	12	2	12	0.23	No
Obesity 1	Module	M00019	Valine/isoleucine biosynthesis,	28	12	2	14	0.24	No
Obesity 1	Module	M00028	Ornithine biosynthesis, glutamate => ornithine	23	11	2	10	0.24	Yes
Obesity 1	Module	M00364	C10-C20 isoprenoid biosynthesis, bacteria	12	6	0	6	0.24	No
Obesity 1	Module	M00527	Lysine biosynthesis, DAP aminotransferase pathway, aspartate => lysine	27	12	2	13	0.24	No
Obesity 1	Module	M00761	Undecaprenylphosphate alpha-L-Ara4N biosynthesis, UDP-GlcA => undecaprenyl phosphate alpha-L-Ara4N	17	0	4	13	0.24	No

[a]Significant based on phylogenetic regression in the same dataset
